# Effects of Acute Psychological and Physiological Stress on Rock Climbers

**DOI:** 10.3390/jcm10215013

**Published:** 2021-10-28

**Authors:** Pamela Villavicencio, Cristina Bravo, Antoni Ibarz, Silvia Solé

**Affiliations:** 1 Master Program Integrative Physiology, University of Barcelona, 08028 Barcelona, Spain; pvillasu13@alumnes.ub.edu; 2 Department of Nursing and Physiotherapy, University of Lleida, 25198 Lleida, Spain; silvia.sole@udl.cat; 3 Research Group of Health Care (GRECS), Institute of Biomedical Research Center, 25198 Lleida, Spain; 4 Department of Cellular Biology, Physiology and Immunology, Faculty of Biology, University of Barcelona, 08028 Barcelona, Spain; tibarz@ub.edu

**Keywords:** stress, cortisol, saliva, anxiety, rock climbers

## Abstract

Background: The aim of this study was to assess the effects that psychological and physiological stressors have on indoor rock climbers, as well as to identify sex differences. Methods: 14 intermediate rock climbers participated in the study, 10 males and 4 females. Mean age was 31 ± 8 years for males and 21 ± 2 years for females. Day 1 consisted of test familiarization and baseline measurements. Day 2 included two test conditions, startle and fatigue, separated by 20 min. In the startle condition, participants had to lead climb a route, and a loud audio stimulus was presented near the top of the climb. In the fatigue condition, participants were required to climb as fast as they could until muscular failure. The competitive state anxiety inventory second review (CSAI-2R) questionnaire was used to assess somatic anxiety, cognitive anxiety, and self-confidence. The four-square step test (FSST) was used to assess motor control, and cortisol levels were acquired via passive drool (PD). Results: Cortisol concentrations were highest in the pre-startle condition (1.72 μg/dL ± 0.66), and values decreased post-startle (1.67 μg/dL ± 0.74) and post-fatigue (1.42 μg/dL ± 0.72). However, cortisol concentrations increased post-startle in females (1.57 μg/dL ± 0.96). Somatic anxiety in males was significantly higher post-startle (16.36 ± 5.54) than pre-startle (14.23 ± 5.09). Females had significantly higher somatic anxiety post-startle (18.00 ± 8.76), and they had lower self-confidence levels (30.00 ± 5.89) than males. Conclusions: There are differences in the way that males and females prepare and respond to stressful situations. Furthermore, time of day may have had a significant impact on cortisol concentrations.

## 1. Introduction

Rock climbing is a complex sport that encompasses both psychological and physiological stressors. Indoor rock climbing has two different climbing techniques: lead and top rope climbing [1,2]. In lead climbing, the climber must attend to the safety rope and clip it into anchors as they make their way up the route. If the climber does not clip the safety rope properly, they will generally fall a short distance. On the other hand, in top rope climbing, the safety rope passes through an anchor at the top of the climb, and the climber does not need to manage it. If a climber falls during a top rope climb, they will sag on the rope. Lead climbing has been associated with increased perceived stress because of the increased mental demand and consequence of falling [2]. However, this does not seem to be the case with advanced rock climbers [1].

When the body’s homeostasis is disrupted, or perceived to be disrupted, the body initiates a stress response. This response includes the activation of the hypothalamic-pituitary-adrenal (HPA) axis [3]. The HPA axis starts with the secretion of corticotropin-releasing hormone (CRH) from the hypothalamus, followed by the release of adrenocorticotropic hormone (ACTH) from the pituitary gland, and finally the release of glucocorticoids from the adrenal glands. Since cortisol can be used as a biological marker of stress, several studies have measured it either via plasma or salivary samples [2,4,5]. The gold standard for salivary cortisol sampling is the passive drool (PD) method, since the effect of flow rate on saliva composition can be discarded [6].

Rock climbing can quickly induce stress due to the fear and anxiety of falling, as well as the elevated cognitive attention it requires to plan movement sequences, recovery positions, speed of the climb, and timing of clipping the safety rope [2]. The amount of perceived stress can also be influenced by the level of expertise of the climber and whether others are present [7,8]. Studies have found that altering the climbing technique to lead climbing increased both subjective anxiety and plasma cortisol concentrations [2]. The peak plasma cortisol concentration is suggested to occur 15–20 min after the stressor, regardless of the climbing technique [1,5,9]. However, one study found that post-climb salivary concentrations were higher immediately after the climb and not 15 min later [10].

Stress can also influence motor skills. Some studies have found that stress can disrupt the accuracy and coordination of movements, as well as posture [11]. Stress also affects the speed of movement in the fight-or-flight response, causing movements to be quicker at the expense of accuracy [11]. These frantic movements lead to decreased success rates, likely because of altered sensory feedback from the lack of haptic feedback [11]. In stressful situations, there may also be impaired cognitive and visuomotor processes that negatively affect motor skills [11].

When this article was published, there was limited data on the role that stress plays in rock climbers, and even more limited literature on differences between sexes. The purpose of this study was to determine the implications that stress has on motor control and cortisol levels in rock climbers and to bridge the gap between psychological and physiological findings. Our hypothesis is that motor control, measured via dynamic balance and coordination with the four-square step test (FSST), will decrease and that cortisol levels will increase, as has been shown in previous studies [2,10].

## 2. Materials and Methods

### 2.1. Participants

A total of 14 participants volunteered to take part in the study, 10 males and 4 females. Mean age, height, and body mass for males was 31 ± 8 years, 176 cm ± 5, and 70 kg ± 6.5, respectively. Mean age, height, and body mass for females was 21 ± 2 years, 166 cm ± 5, and 59 kg ± 2.9, respectively. Participant information is reported in Table 1 and Table 2. The study took place at an indoor rock-climbing gym. All participants were intermediate climbers, with a minimum skill level of 6c. They had no injuries or underlying medical conditions and had low to moderate stress levels, as measured by the Perceived Stress Scale (PSS) [12]. Participants completed an informed consent form after a thorough explanation of the study and after completing a physical activity readiness questionnaire (PAR-Q) [13].

### 2.2. Competitive State Anxiety Inventory Second Review (CSAI-2R) Questionnaire

The CSAI-2R consists of 17 items that are scored on a Likert scale from 1 to 4, and the combined scores result in a final score on each of the 3 subscales (somatic anxiety, cognitive anxiety, and self-confidence). The European Spanish version of the CSAI-2R consists of 18 items [14].

### 2.3. Four Square Step Test (FSST)

The FSST is a way to measure dynamic balance, stability, and coordination [15]. It requires two sticks to be placed on the floor so that they form a “plus sign”, and the participants must step in each square in a set sequence. There are two trials, and the best time is recorded [16].

### 2.4. Procedure: Day 1

Day 1 of the intervention consisted of anthropometric measurements, procedure familiarization, and strength tests. Participants completed an easy route with the top rope technique. Prior to the climb, they underwent a hand grip test with a Saehan Spring Hand^®^ dynamometer (Saehan Corporation, Changwon 630-728, South Korea) and completed the FSST and the CSAI-2R.

During the climb, participants wore a Polar A300 watch and Polar H10 heart rate monitor (Polar Electro^®^, Kempele, Finland). They were instructed to climb at their normal pace. After the climb, they completed the hand grip test, the FSST, and the CSAI-2R again.

### 2.5. Day 1 Strength Tests

The participants finished day 1 with two strength tests: a maximum pull-up test and a bent-arm hang test. These tests were used to assess shoulder power and endurance, which has been shown to be the primary determinant for success in rock climbing [17]. The tests were included to provide objective measurements of participants’ physical abilities. The pull-up test was performed with a pronated grip, and 1 repetition was considered as chin over the bar and full elbow extension. The bent-arm hang test was measured as the maximum time the participants could hang with elbows at 90° in the pronated grip position. Results are presented in Table 1 and Table 2.

### 2.6. Day 1 Passive Drool Instructions

At the end of day 1, participants were given 9 Salivette^®^ Cortisol vials (Sarstedt AG & Co., Nümbrecht, Germany) without the synthetic swab, and instructed to take baseline salivary samples for three days at 8:00 a.m., 11:00 a.m., and 2:00 p.m. Participants were given clear written and verbal instructions on the PD method.

### 2.7. Procedure: Day 2

Day 2 consisted of two different test conditions, separated by a recovery period of 20 min. The first test condition was the startle condition, and it took place at 8:00 a.m. The second condition was the fatigue condition, and it took place at 8:30 a.m. The order of the test conditions was fixed to eliminate the effects of physiological and psychological fatigue in the startle condition. Since the climbing route of the startle condition was more difficult and required a high cognitive, physical, and tactical demand, this condition was performed first. Before climbing, participants had their resting blood pressure taken, and they completed the FSST and CSAI-2R. They provided saliva samples via passive drool, and their 3 min average pre-climb heart rate (Pre-HR_3min_) was recorded.

The startle condition consisted of a loud stimulus and the added stress of having to lead climb a route that gradually increased in difficulty. There were two possible routes in this condition: either a route that progressed in difficulty from 6b to 6c+ or from 6b to 7b. When the participants were preparing to make a key jump to a more difficult section, an air horn was used to startle them (Goodmark^®^, Llantarnam, UK). Three of the final participants had an alternate audio stimulus due to lack of gas in the air horn. With these final participants, the investigator hit a pan with a wooden spatula and screamed.

During the climb, the climb duration, success/fail, HR at the start of the climb (HR_start_), HR average (HR_avg_), and peak HR (HR_peak_) were recorded. Immediately after the climb, blood pressure was measured, and participants completed the FSST and CSAI-2R. The climbs were filmed with a Samsung N363 digital camcorder (Samsung Group, Hwaseong, South Korea). Fifteen minutes after the startle, post-climb salivary samples were taken. The time for HR to return to pre-climb levels (Post-HR_recovery_) was also recorded.

In the fatigue condition, participants had to top rope climb a predetermined route as fast as they could and as many times as they could, until muscular failure. There were three routes that varied in difficulty (6b, 6c+, 7b), and the specific route assigned to each participant was based on their self-reported skill level. Time splits of each climb, the number of falls until fatigue, and HR_start_, HR_avg_, and HR_peak_ were all recorded. Each climb was filmed. Immediately post-climb, participants had their blood pressure measured, and they completed the FSST and CSAI-2R. Fifteen minutes post-climb, they completed a final salivary sample. Their post-HR_recovery_ time was also recorded.

### 2.8. Saliva Samples

The PD method requires participants to sit with his or her head flexed forward while saliva passively drips into a container [18]. Participants stored the samples in their refrigerator (4 °C) until day 2, where they brought the samples to the rock-climbing gym. Approximately 3 mL of saliva was collected.

Salivary cortisol samples were stored at −80 °C until analysis. They were then centrifuged at 2500× *g* for 10 min, and 1.5 mL of the separated samples was placed in Eppendorf microtubes (Starsledt Akhengesellshaft & Co., Nümbrecht, Germany). Salivary cortisol was measured with a Cortisol Saliva Enzyme-Linked Immunosorbent Assay (ELISA) procedure (IBL International GMBH, Hamburg, Germany).

### 2.9. Statistics

All analyses were performed using the statistical package IBM^®^ SPSS^®^ Statistics Software (SPSS) version 26. Results are expressed as mean ± standard deviation. After verifying that all values were within the normal range, *T* tests were performed to compare the mean values of different conditions. A one-way ANOVA analysis was used to compare the variables measured between sexes and between test conditions. Pearson’s correlation was utilized to analyze the relationship between time to fatigue and body weight, as well as the number of pull-ups and cortisol levels. Differences were considered significant at *p* < 0.05.

## 3. Results

### 3.1. Cortisol

No outliers in cortisol concentrations were identified; however, values that were not within the reportable range of 0.015–3.00 μg/dL were discarded, as indicated by the Cortisol Saliva ELISA kit (IBL International GMBH, Germany). Baseline cortisol concentrations were highest at 8:00 a.m., with average values of 0.71 μg/dL ± 0.35. There were significant differences between cortisol concentrations at 8 a.m. for the three baseline measurements in males (0.78 μg/dL ± 0.47, *p* = 0.00; 0.59 μg/dL ± 0.43, *p* = 0.002; 0.80 μg/dL ± 0.56, *p* = 0.003), as well as when compared to pre-startle (1.85 μg/dL ± 0.70, *p* = 0.000), post-startle (1.73 μg/dL ± 0.67, *p* = 0.000), and post-fatigue (1.44 μg/dL ± 0.61, *p* = 0.000). There were no significant differences in cortisol concentrations in males when comparing the three test conditions (1.85 μg/dL ± 0.70, 1.73 μg/dL ± 0.67, 1.44 μg/dL ± 0.61).

**Figure 1:**
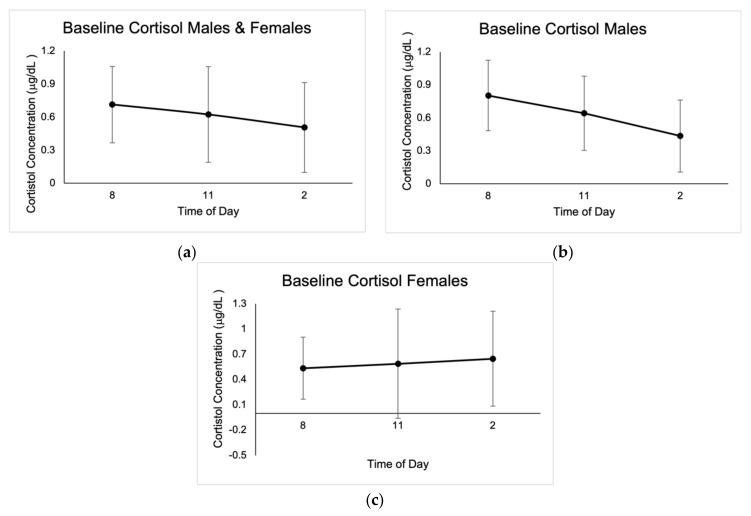
Mean baseline salivary cortisol concentrations expressed in μg/dL ± SD from the three days of sampling at 8:00 a.m., 11:00 a.m., and 2:00 p.m. (**a**) Results for males and females. (**b**) Results for males. (**c**) Results for females.

**Figure 2:**
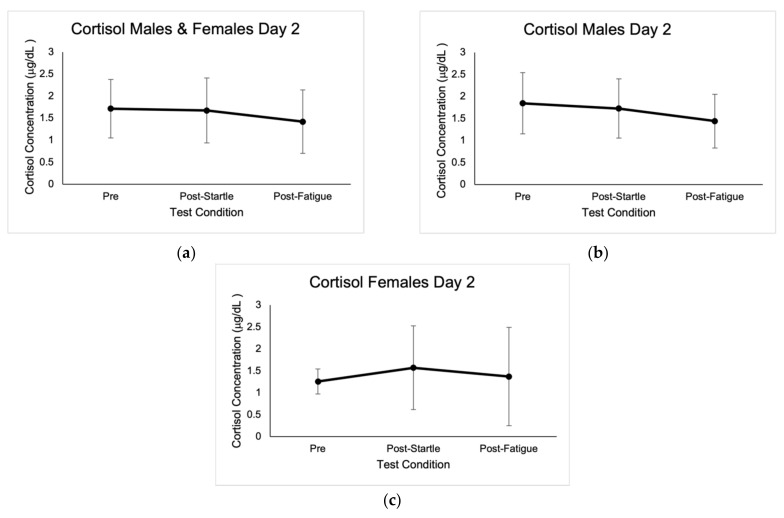
Mean cortisol concentrations expressed in μg/dL ± SD from the three test conditions of day 2. (**a**) Results for males and females. (**b**) Results for males. (**c**) Results for females.

Post-startle cortisol concentrations were significant (1.57 μg/dL ± 0.96, *p* = 0.046) for females when compared with pre-test levels (1.26 μg/dL ± 0.29), and there were also significant differences (*p* = 0.043) between female pre-startle (1.26 μg/dL ± 0.29) and post-fatigue (1.37 μg/dL ± 1.12). There were significant differences (*p* = 0.050) in males and females between pre-startle (1.72 μg/dL ± 0.66) and post-fatigue (1.42 μg/dL ± 0.72) cortisol levels. Cortisol baseline concentrations are shown in Figure 1, and concentrations in the different test conditions are shown in Figure 2.

There were no significant differences between sexes in cortisol concentrations in the pre-startle (1.85 μg/dL ± 0.70, 1.26 μg/dL ± 0.29), post-startle (1.73 μg/dL ± 0.67, 1.57 μg/dL ± 0.96), and post-fatigue (1.44 μg/dL ± 0.61, 1.37 μg/dL ± 1.12) conditions. Based on the ANOVA F analysis, there was a positive correlation between number of pull-ups and pre-test cortisol concentrations (*p* = 0.008, r = 0.814, R^2^ = 0.663, CI = 95%).

### 3.2. Heart Rate

Heart rate followed the expected pattern during both climbs. During the startle climb, values increased progressively as the climb went on. During the fatigue climb, heart rate values increased throughout the climb, and the initial heart rate was higher for each subsequent climb.

### 3.3. FSST

There were no significant differences between FSST values baseline pre-climb day 1 (3.62 s ± 0.75) and post-startle day 2 (3.09 ± 0.62). There were significant differences (*p* = 0.006) between FSST scores baseline post-climb day 1 (3.45 s ± 0.61) and post-startle day 2 (3.09 s ± 0.62), and significant differences (*p* = 0.002) between males’ baseline post-climb day 1 (3.42 s ± 0.53) and post-fatigue day 2 (2.84 s ± 0.43) scores. Results are shown in Table 3, Table 4 and Table 5.

### 3.4. Anxiety and Self-Confidence

There were significant differences (*p* = 0.019) in somatic anxiety pre-startle (14.23 ± 5.09) and post-startle (16.36 ± 5.54) in males, as well as significant differences (*p* = 0.035) between male and female self-confidence levels pre-startle (35.08 ± 4.94, 30.00 ± 5.29). There were also significant differences (*p* = 0.022) in self-confidence post-fatigue between sexes (34.43 ± 5.88, 29.00 ± 4.76). Results are shown in Table 3, Table 4 and Table 5.

### 3.5. Grip Strength

There were significant differences (*p* = 0.012) between baseline left-hand grip strength (48.60 kg ± 9.36) and post-fatigue left-hand grip strength (36.46 kg ±8.63). Males had significant differences (*p* = 0.035) between baseline left-hand grip strength (52.27 kg ± 6.94) and post-fatigue left-hand grip strength (35.35 ± 14.74). There were differences in female baseline left-hand grip strength (38.50 kg ± 7.94) and post-fatigue left-hand grip strength (29.25 kg ± 2.36), although not significant (*p =* 0.058). Males also had significant (*p =* 0.00) differences in pre-startle (50.55 ± 10.48) and post-startle (51.05 ± 8.11) right--hand grip strength. Results are reported in Table 3, Table 4 and Table 5.

### 3.6. Fatigue

There was an inverse correlation between time to fatigue and body weight (CI = 95%, r = 0.606, *p =* 0.025). There were significant differences (*p =* 0.022) between sexes: males reached muscular failure after 282.39 s ± 48.20, and females after 367.51 s ± 70.21.

## 4. Discussion

The results indicate that physical and psychological stress affects males and females in different ways and that cortisol concentrations are strongly affected by time of day. Salivary samples were utilized in this study because since cortisol follows a circadian rhythm, with the highest values occurring 20–40 min after waking, we thought it beneficial to obtain a baseline secretion curve for comparison with the rest of the values [18,19]. Baseline cortisol concentrations followed a normal diurnal pattern, with the highest values occurring at 8:00 a.m.

It is possible that the variations in concentrations between the 8:00 a.m. samples of each day were due to individual error or individual variation in diurnal cortisol slope (DCS). Cortisol concentrations can be easily affected by acute stressors, age, sex, nutrition, sleep, hydration, physical activity, and circadian rhythm [4,9,18]. Salivary composition can also be affected by countless factors, including circadian rhythm, age, sex, smoking, diet, and medications [4]. Since external factors of the participants’ day to day were not accounted for, it is possible that variations in these variables altered their DCS. In addition, it is possible that participants did not take the samples at the same time for each of the three baseline days. Although these may have been small variations, it may have been enough to affect the DCS substantially—especially in the waking hours [19,20]. Variations in sampling time may have also been due to difficulty in saliva production. Some participants reported spending 20 min in the PD position to produce sufficient saliva. This may have further delayed the time of day that the sample was obtained, thus influencing cortisol levels. This may have also been a factor on day 2, since some participants took substantially longer to produce enough saliva pre-startle, post-startle, and post-fatigue. Although the pre-startle samples were taken at 8:00 a.m., and the post-startle and post-fatigue samples were taken shortly after, time of day may have profoundly impacted the variance in salivary cortisol levels. Since the fatigue climb was the last test condition, this could explain the decrease in cortisol levels in males and females. Instead of obtaining baseline samples at 8:00 a.m., 11:00 a.m., and 2:00 p.m., perhaps obtaining samples at 8:00 a.m. and 9:00 a.m. would have been a better comparison for this study. Moreover, lead climbing and the auditory stimulus used may not have been strong enough stressors to provoke changes in cortisol levels in males due to their level of experience and more advanced skill level [1].

The 8:00 a.m. pre-startle cortisol levels were higher in both males and females when compared to their respective baseline 8:00 a.m. cortisol levels. This may have been due to an anticipatory cortisol response that primes the central nervous system [21]. This anticipatory response provides some insight into the relationship between psychological stress and physiological responses, as well as highlights the significance of psychobiological processes that occur prior to a stressor. It is possible that this neuroendocrine response was activated when instructions for the startle climb were provided. This would suggest that the stress (and increase in cortisol) that individuals experienced was triggered by their emotional and cognitive representations of what they thought would occur during the climb [21].

Females may have experienced a peak in cortisol levels post-startle because the relative difficulty of the climb may have been higher for them. Female participants were not as comfortable with the lead climbing technique, and this lack of confidence, in addition to the sustained isometric contractions and increasing difficulty of the climb, may have contributed to a peak in cortisol levels post-startle. This is supported by the significant differences in self-confidence between males and females prior to the startle climb.

Increased somatic anxiety post-startle in males may have been due to the added stress of lead climbing. Other studies have had similar findings, noting that participants had increased somatic anxiety when they had to lead climb a route, compared to top rope climbing [2]. Sex differences in somatic anxiety post-startle may be an indicator of differences in male and female responses to stress. There is evidence from functional magnetic resonance imaging (fMRI) that women are more attuned to negative stimuli and that they respond more rapidly to negative stimuli [22]. These sex differences may also explain differences in the self-reported self-confidence post-fatigue climb.

FSST times may have been faster post-startle because of heightened somatic anxiety and focus, due to the fight-or-flight response. It is also possible that the results were influenced by test familiarization and decreased anxiety of social judgment. In day 1, the FSST trials were carried out when the rock-climbing gym was open to the public. Therefore, there were other climbers present that served as an “audience” to the participants in the study. The participants may have also had difficulty focusing on the task at hand because of the various distractions in the gym. The fact that the participants did not know what to expect, that it was their first time performing the FSST, that there was an audience, and that their focus could have been affected, may have all contributed to slower day 1 scores.

It could be that average FSST times did not decrease post-fatigue because the value that was used to indicate fatigue was forearm muscle failure. It may be that although the forearm musculature fatigued to failure, focus and lower limb coordination did not decline. It is also worth noting that three participants were not able to complete the test post-fatigue, due to poor coordination and unsuccessful execution of the sequence.

## 5. Conclusions

Our results show that cortisol concentrations follow a normal standard curve, irrespective of the test condition. Cortisol samples were taken 15 min after the stressor, and values were lower post-startle and post-fatigue when compared to the pre-test. It may be that the stressors used in this study were not enough to provoke a stressful situation in the climbers of this study or that higher values were presented immediately after the climb and not 15 min later. Future studies should compare the cortisol response immediately after the stimulus, as well as 15 min later, to determine when the true peak in cortisol occurs. Studies should also look at ways to reduce the amount of time spent in saliva sampling, since extended sampling time may have profoundly affected cortisol levels.

There seem to be differences in the way that males and females psychologically prepare and react to stressful situations. It is difficult to draw conclusions from this sample because a major limitation was the number of participants, especially females. Evidently, there are countless factors that can influence the stress response during climbing, as well as several variables that can serve as indicators of the demand of the climb. Future studies should also take into consideration the biomechanical and strategic changes that occur with increased psychological and physiological stress. This can be done by analyzing video footage and utilizing electromyography (EMG) to determine premotor time and reaction time, as well as changes in muscle activity. Blood samples can also be taken to look at the impact that acute stressors have on biomarkers of oxidative stress, as well as on biomarkers that are suggested to be related to anxiety [4].

## Figures and Tables

**Table 1:** Male participant information.

Males	*N*	Min	Max	Mean	SD
Age	10	17	43	30.7	8.49
Height	10	165	180	174.4	4.77
Weight	10	61.6	83.9	70.3	6.52
Years climbing	10	2	25	9.89	8.49
Skill level	10	6b	8c		
Max pull-ups	10	8	29	17	6.89
Bent-arm hang	10	27	76	48.6 s	14.83 s

**Table 2:** Female participant information.

Females	*N*	Min	Max	Mean	SD
Age	4	19	24	21	2.45
Height (cm)	4	161	173	165.75	5.5
Weight (kg)	4	55.7	62	59	2.95
Years climbing	4	2	8	4.75	2.5
Skill level	4	6b	7c+		
Max pull-ups	4	7	18	14	4.83
Bent-arm hang	4	30	48	38	8.91 s

**Table 3:** Summary of results for males and females. Significant differences (*p* = 0.003) in somatic anxiety post-startle between males and females. Significant differences (*p* = 0.019) between male and female self-confidence values pre-startle and post-fatigue (*p* = 0.02). Significant differences (*p* = 0.006) in FSST times between baseline post-climb and post-startle. Significant differences (*p* = 0.012) between baseline pre-climb left-hand grip strength and post-fatigue left-hand grip strength.

Variables	Baseline Pre-Climb	Baseline Post-Climb	Pre-Startle	Post-Startle	Pre-Fatigue	Post-Fatigue
Somatic Anxiety	15.50 ± 4.49	17.25 ± 5.64	14.23 ± 5.09	16.36 ± 5.54	15.33 ± 5.03	16.43 ± 5.26
Cognitive Anxiety	16.14 ± 5.68	13.50 ± 4.52	13.54 ± 6.64	13.14 ± 4.69	12.67 ± 4.62	12.00 ± 3.84
Self-Confidence	35.43 ± 5.57	34.50 ± 5.13	35.08 ± 4.94	33.14 ± 7.18	33.33 ± 6.57	34.43 ± 5.88
FSST	3.62 s ± 0.75	3.45 s ± 0.61	3.19 s ± 0.52	3.09 s ± 0.62	2.89 s ± 0.48	2.84 s ± 0.43
Grip Strength Right	46.57 kg ± 10.20	49.90 kg ± 11.38	46.64 kg ± 11.32	46.43 kg ± 10.34	41.15 kg ± 19.18	395.17 ± 13.42
Grip Strength Left	48.60 kg ± 9.36	48.03 kg ± 12.17	46.14 kg ± 9.88	44.64 kg ± 9.58	39.74 kg ± 18.51	33.47 kg ± 12.44

**Table 4:** Summary results for males. Significant differences (*p* = 0.019) in somatic anxiety pre- and post-startle in males. Significant differences (*p* = 0.002) between FSST baseline post-climb and post-fatigue scores. Significant differences (*p* = 0.035) between baseline pre-climb left-hand grip strength and post-fatigue left-hand grip strength. Significant differences (*p* = 0.00) between pre-startle and post-startle right-hand grip strength.

Variables	Baseline Pre-Climb	Baseline Post-Climb	Pre-Startle	Post-Startle	Pre-Fatigue	Post-Fatigue
Somatic Anxiety	14.50 ± 4.35	17.50 ± 6.07	14.23 ± 5.09	16.36 ± 5.54	15.33 ± 5.03	16.43 ± 5.26
Cognitive Anxiety	15.80 ± 6.49	13.75 ± 5.18	13.54 ± 6.64	13.14 ± 4.69	12.67 ± 4.62	12.00 ± 3.84
Self-Confidence	37.20 ± 3.55	36.50 ± 3.96	35.08 ± 4.94	33.14 ± 7.18	33.33 ± 6.57	34.43 ± 5.88
FSST	3.71 s ± 0.82	3.42 s ± 0.53	3.19 s ± 0.52	3.09 s ± 0.62	2.88 s ± 0.48	2.84 s ± 0.43
Grip Strength Right	49.95 kg ± 9.22	53.59 kg ± 10.77	50.55 kg ± 10.48	51.05 kg ± 8.11	46.52 kg ± 18.55	37.91 kg ± 15.31
Grip Strength Left	52.27 kg ± 6.94	51.91 kg ± 11.40	50.40 kg ± 7.99	48.15 kg ± 8.88	45.14 kg ± 17.92	35.35 kg ± 14.74

**Table 5:** Summary results for females.

Variables	Baseline Pre-Climb	Baseline Post-Climb	Pre-Startle	Post-Startle	Pre-Fatigue	Post-Fatigue
Somatic Anxiety	18.00 ± 4.32	16.75 ± 5.50	15.33 ± 8.39	18.00 ± 8.76	16.25 ± 6.85	17.25 ± 8.46
Cognitive Anxiety	17.00 ± 3.46	13.00 ± 3.46	15.33 ± 9.24	13.50 ± 5.74	13.50 ± 4.73	11.00 ± 1.15
Self-Confidence	31.00 ± 7.75	30.50 ± 5.26	30.00 ± 5.29	30.00 ± 5.89	29.00 ± 5.77	29.00 ± 4.76
FSST	3.41 s ± 0.58	3.52 s ± 0.87	3.34 s ± 0.76	3.60 s ± 0.95	3.09 s ± 0.76	3.13 s ± 0.68
Grip Strength Right	37.25 kg ± 6.65	39.75 kg ± 5.56	36.88 kg ± 6.91	34.88 kg ± 4.13	30.41 kg ± 17.76	29.00 kg ± 4.69
Grip Strength Left	38.50 kg ± 7.94	37.38 kg ± 7.18	35.50 kg ± 4.49	35.88 kg ± 4.17	28.94 kg ± 16.52	29.25 kg ± 2.36

## Data Availability

Not applicable.
